# Relationship between overall survival and progression-free survival with 18 F-FDG PET/CT metabolic parameters in metastatic breast cancer patients treated with CDK 4/6 inhibitors

**DOI:** 10.1007/s11845-026-04505-w

**Published:** 2026-06-17

**Authors:** Ogün Bülbül, Esra Aydin, Demet Nak

**Affiliations:** 1 Faculty of Medicine, Department of Nuclear Medicine, Erciyes University, Kayseri, 38039 Turkey; 2 Faculty of Medicine, Department of Medical Oncology, Recep Tayyip Erdoğan University, Rize, 53020 Turkey; 3 Faculty of Medicine, Department of Nuclear Medicine, Recep Tayyip Erdoğan University, Rize, 53020 Turkey

**Keywords:** CDK 4/6 inhibitors, Metastatic breast cancer, 18F-FDG PET/CT, MTV/TLG, Survival

## Abstract

**Background:**

Combining CDK 4/6 inhibitors with endocrine therapy (ET) extends progression-free survival (PFS) and overall survival (OS) in hormone receptor (HR) positive, human epidermal growth factor receptor-2 (HER2) negative metastatic breast cancer. We aimed to explore the relationship between metabolic tumor volume (MTV), total lesion glycolysis (TLG), and PFS/OS in these patients.

**Methods:**

MTV and TLG were measured from baseline 18 F-fluorodeoxyglucose (18-FDG) positron emission tomography/computed tomography (PET/CT) of 87 HR-positive/HER2-negative metastatic breast cancer patients and their effects on survival were analyzed using Kaplan-Meier methods.

**Results:**

The median OS was 42.0 months for patients with MTV < 121.7 cm³ and 24.6 months with ≥ 121.7 cm^3^ (*p* = 0.001). The median OS was 40.3 months for patients with TLG < 732.4 g and 24.6 months with ≥ 732.4 g (*p* = 0.006). The median OS for patients with an ECOG score of 0 was 53.6 months, while for those with an ECOG score of 1–2, it was 26.4 months (*p* = 0.005). Age ≥ 60 years (HR = 5.11, *p* = 0.002) and ECOG score > 0 (HR = 3.38, *p* = 0.011) are independent predictors of PFS.

**Conclusions:**

Baseline 18 F-FDG PET/CT may provide prognostic insights in HR+/HER2- metastatic breast cancer. High MTV and TLG are associated with poorer OS in univariate analyses but not in multivariable analyses. ECOG score > 0 and age ≥ 60 years are independent predictors of PFS.

## Introduction

Cyclin-dependent kinase (CDK) 4/6 inhibitors stop cell division by blocking the transition from the G1 phase to the S phase of the cell cycle [1]. Several studies have demonstrated that combining CDK 4/6 inhibitors with endocrine therapy improves both progression-free survival (PFS) and overall survival (OS) in patients with hormone receptor (HR)-positive, human epidermal growth factor receptor 2 (HER2)-negative metastatic breast cancer [2–8].

Circulating tumor DNA has proven to be promising for predicting early progression under CDK 4/6 inhibitors or for monitoring treatment response [9]. Additionally, overexpression of miR‑21, ‑34a, ‑193b, ‑200a, and ‑200b in plasma was associated with progression detected through liquid biopsy [10]. However, more accessible, cost-effective, and reliable biomarkers are still needed to optimize the use of CDK 4/6 inhibitors. 18 F-fluorodeoxyglucose (18-FDG) positron emission tomography/computed tomography (PET/CT) is a molecular imaging technique that offers both anatomical and functional information. Routine use of 18 F-FDG PET/CT is not advised for patients with breast cancer [11]. However, 18 F-FDG PET/CT aids in disease management for patients with locally advanced breast cancer or suspicious findings on other imaging tests [12]. Metabolic parameters derived from 18 F-FDG PET/CT have also been shown to predict hormone receptor status, HER2 status, and survival outcomes in newly diagnosed breast cancer [12–14]. In patients with metastatic breast cancer treated with CDK 4/6 inhibitors as second-line therapy, lower SUVmax was associated with longer PFS [15], while in patients treated as first-line therapy, baseline metabolic tumor volume (MTV) was associated with PFS but not OS [16]. In another study [17], it was reported that MTV or TLG could not predict PFS in patients who received CDK 4/6 inhibitors as first- or second-line therapy. In this study, we aimed to determine the relationship between MTV and total lesion glycolysis (TLG), which indicate tumor burden, and both PFS and OS in HR(+)/HER2(-) metastatic breast cancer patients treated with CDK 4/6 inhibitors plus endocrine therapy as first-line treatment.

## Methods

This study received approval from the local ethics committee (Decision no: 2025/368, decision date: 21.08.2025). The requirement for informed consent was waived by the ethics committee. This study was conducted according to the principles of the Declaration of Helsinki.

### Patients

**Fig. 1:**
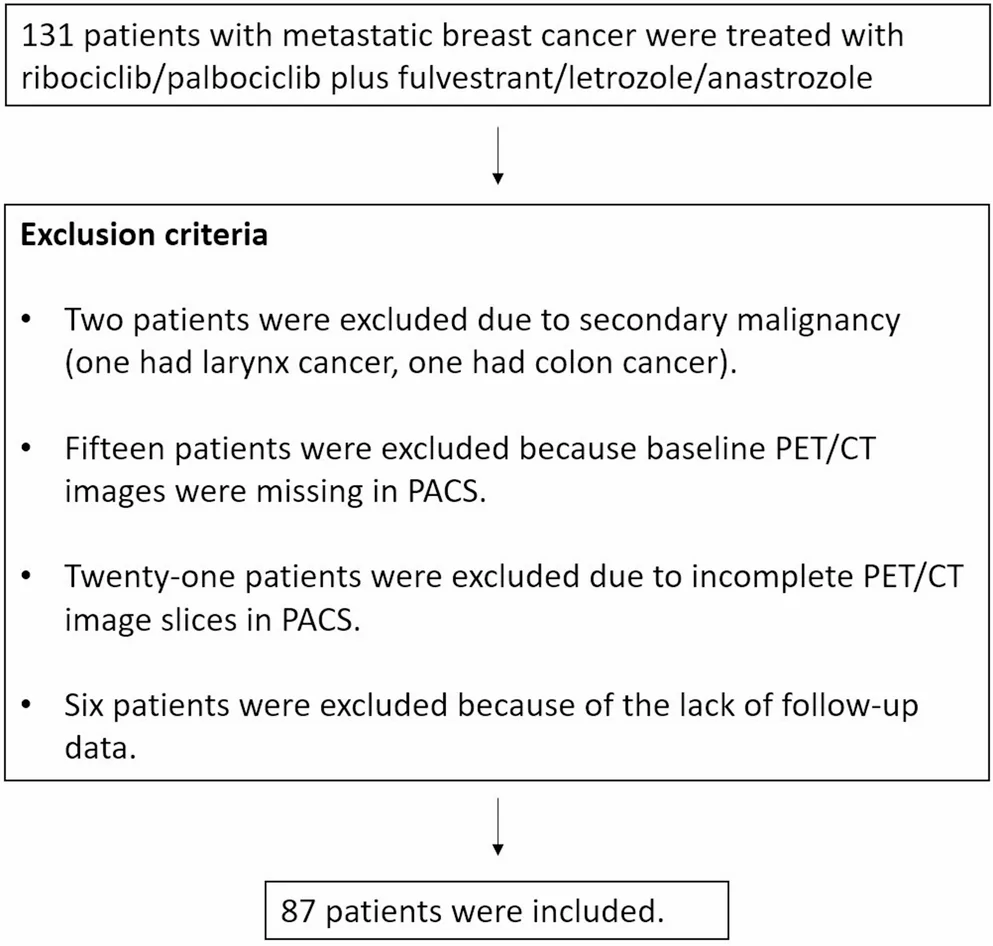
Patient exclusion criteria

We retrospectively identified 131 female patients with HR+/HER2- metastatic (de novo metastatic or relapsed with metastases) invasive breast cancer who received ribociclib or palbociclib in combination with fulvestrant, letrozole, or anastrozole as their first-line treatment at our hospital between 2019 and 2024. Inclusion criteria were: (1) Patients aged 18 or older, (2) Patients who underwent pretreatment PET/CT. Exclusion criteria were: (1) Inaccessibility of medical records during follow-up, (2) PET/CT performed at another center, (3) Missing or incomplete PET/CT images in Picture Archiving Communication Systems (PACS), and (4) Presence of a secondary malignancy. Of the 131 eligible patients screened, 87 who met the inclusion and exclusion criteria were included in the study (Fig. 1).

### 18 F- FDG PET/CT protocol and imaging analysis

All patients underwent PET/CT scans using a Siemens mCT 20. They fasted for at least six hours before imaging. Patients with fasting blood glucose levels below 200 mg/dl received an intravenous injection of 3.7 MBq (0.1 mCi)/kg of 18 F-FDG. They remained in a darkened room for 45–60 min before PET/CT images were acquired from the vertex to the mid-thigh. PET images were acquired with a time of two minutes per bed position, and iterative reconstruction was performed using the ordered subset expectation maximization method (20 subsets, two iterations). Non-contrast CT images were obtained for attenuation correction and anatomical correlation (120 kVp, 50 mAs, 3 mm slice thickness).

**Fig. 2:**
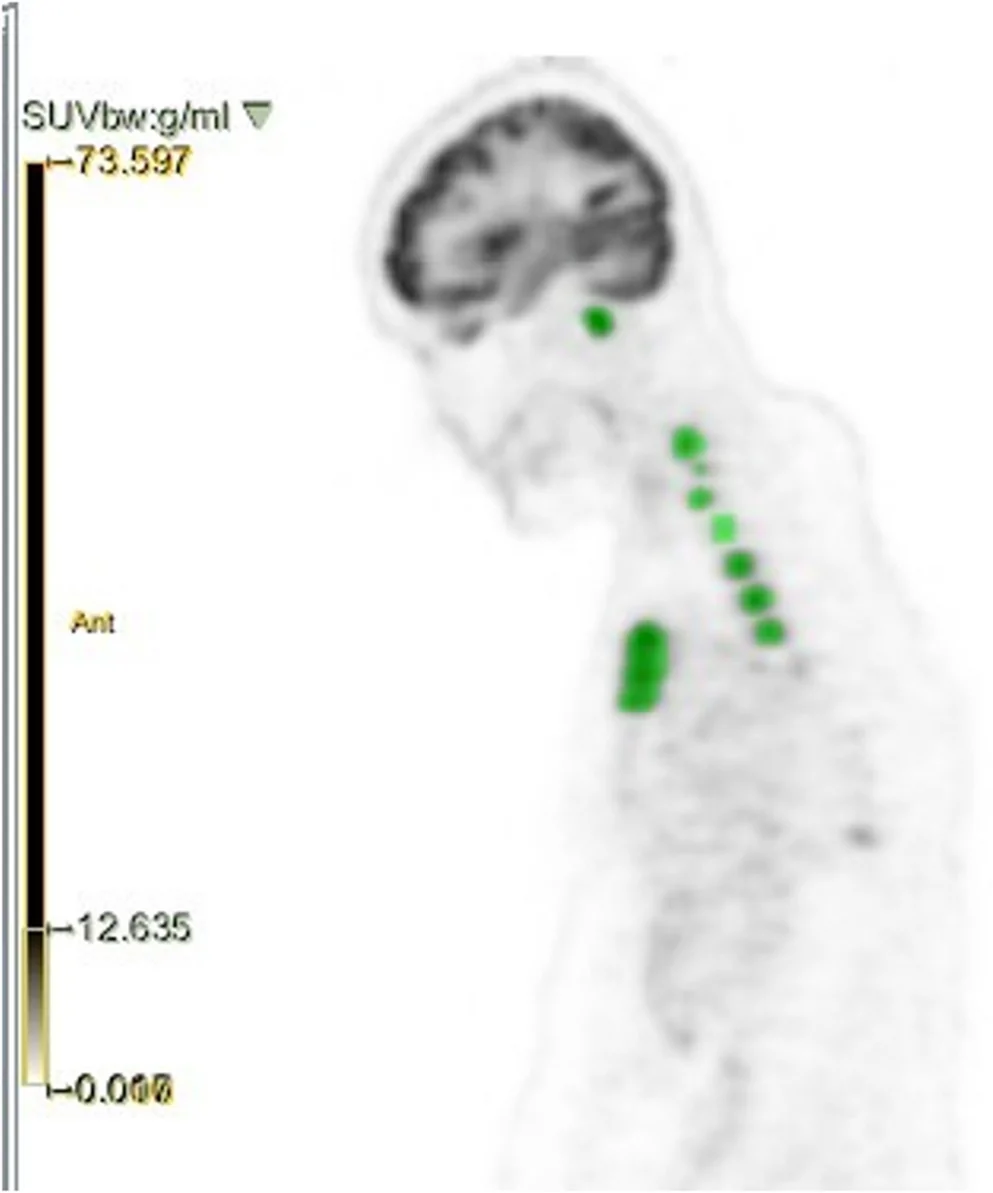
Measurement of MTV and TLG from metastatic lesions using semi-automatic segmentation

PET/CT images were downloaded from PACS. Freely available LIFEx software (version 25.06.1) was used for MTV and TLG calculations [18]. Initially, lesions with an SUVmax > 2.5 that were considered malignant based on correlation with CT findings (primary tumor/metastasis) were identified using semi-automatic segmentation (Fig. 2). MTV was calculated using a threshold of 40% of SUVmax, in accordance with commonly adopted methodology. Two nuclear medicine physicians, each with nine years of experience, jointly reviewed all segmentations. Manual segmentation was performed when automatic segmentation failed to identify metastatic foci. LIFEx software automatically calculated the TLG (TLG = MTV x SUVmean).

### Ribociclib and palbociclib treatment protocol

Ribociclib was taken orally at 600 mg/day for three weeks, then a one-week break. If side effects appeared, the dose was reduced to 400 mg/day or 200 mg/day. Palbociclib was taken orally at 125 mg/day for three weeks, followed by a one-week break. If side effects occurred, the dose was reduced to 100 mg/day or 75 mg/day.

### Determination of progression-free survival and overall survival

PFS was defined as the time from the start of ribociclib/palbociclib treatment to the first disease progression, if it occurred, or to death if there was no progression. OS was the time until death or the last follow-up date for surviving patients. To determine PFS, results from radiological [computed tomography (CT), magnetic resonance imaging (MRI), and ultrasonography (US)] or molecular (bone scan or PET/CT) examinations were reviewed from medical records.

### Statistical analysis

IBM SPSS Statistics (Version 25) was used for statistical analysis. Categorical variables were presented as counts and percentages. Continuous variables were tested for normal distribution using the Kolmogorov-Smirnov test. Continuous variables that followed a normal distribution were expressed as mean ± standard deviation, while those that did not follow a normal distribution were expressed as median (minimum-maximum).

The frequency of categorical variables in patients who achieved and failed one- and three-year PFS/OS was analyzed using chi-square analysis, with Fisher’s exact test applied when necessary. Differences in MTV and TLG values between patients who achieved and failed one- and three-year progression-free and overall survival were assessed using the Mann-Whitney U test. Threshold values of the MTV and TLG for predicting PFS and OS were determined with ROC curve analysis. Additionally, the relationship between MTV and TLG and PFS/OS was evaluated using Kaplan-Meier analysis and the log-rank test. Univariate and multivariate Cox regression analyses were conducted to identify independent predictors of PFS and OS. Variables with *p* < 0.2 in univariate analysis were included in the multivariate analysis.

In all statistical tests, a p-value of less than 0.05 was considered indicative of statistical significance.

## Results

### Patient characteristics

The mean age of the 87 patients included in the study was 58 years. The median time between the PET/CT scan and the start of ribociclib/palbociclib treatment was 26 days (range, 1-125 days). The majority of patients (79%) had invasive ductal carcinoma, grade 2 tumors (77%), and stage IV cancer at diagnosis (60%). Nearly half of the patients (51%) had an ECOG score of 0. The majority (63%) had no visceral metastases. Most patients (71%) were treated with ribociclib, and letrozole was the most commonly used endocrine therapy in combination (69%). Median follow-up was 25.4 months (range 2.5–66.5). During follow-up, 44 patients (51%) died, and 47 patients (54%) experienced disease progression. Sixty-seven patients (77%) reached one-year PFS, and 22 patients (25%) reached three-year PFS. Seventy-four patients (85%) reached one-year OS, and 27 patients (31%) reached three-year OS. Detailed patient characteristics are provided in Table 1.

**Table 1:** Detailed characteristics of patients

Variables		
Age, mean ± SD	58 ± 14	
Histopathological subtype, n (%)	**IDC**	68 (79)
	**ILC**	14 (16)
	**IDC + ILC**	2 (2)
	**Others**	(3)
History of breast surgery, n (%)	**Present**	42 (48)
	**Absent**	44 (51)
	**N/A**	1 (1)
Ki-67, median (minimum-maximum)	18 (10–60)	
Grade, n (%)	**Grade 1**	6 (7)
	**Grade 2**	67 (77)
	**Grade 3**	9 (10)
	**N/A**	5 (6)
TNM stage at diagnosis, n (%)	**Stage 1**	2 (2)
	**Stage 2**	14 (16)
	**Stage 3**	17 (20)
	**Stage 4**	52 (60)
	**N/A**	2 (2)
CDK 4/6 inhibitor agent, n (%)	**Ribociclib**	62 (71)
	**Palbociclib**	25 (29)
When did the patient become metastatic? n (%)	**De novo**	53 (61)
	**After relapse**	34 (39)
ECOG performance status, n (%)	**0**	44 (51)
	**1**	30 (35)
	**2**	7 (8)
	**3–4**	0 (0)
	**N/A**	6 (6)
Metastatic areas, n (%)	**Bone ± soft tissue**	55 (63)
	**Only visceral organs**	4 (5)
	**Bone + visceral organs**	28 (32)
MTV (mL), median (minimum-maximum)	105,4 (0-3439,5)	
TLG (g), median (minimum-maximum)	381,8 (0-40125,1)	
Additional hormone therapy, n (%)	**Fulvestrant**	25 (29)
	**Letrozole**	60 (69)
	**Anastrozole**	1 (1)
	**N/A**	1 (1)
Bone-modifying agent, n (%)	**Denosumab**	44 (51)
	**Zoledronic acid**	29 (33)
	**No**	14 (16)
Adjuvant/neoadjuvant chemotherapy, n (%)	**Yes**	48 (55)
	**No**	39 (45)
Radiotherapy, n (%)	**Yes**	31 (36)
	**No**	54 (62)
	**N/A**	2 (2)
Previous hormonal therapy, n (%)	**Yes**	47 (54)
	**No**	40 (46)

*SD* standard deviation, *IDC* invasive ductal carcinoma, *ILC* invasive lobular carcinoma, *N/A* not available, *TNM* tumor, node, and metastasis, *MTV* metabolic tumor volume, *TLG* total lesion glycolysis

### Analysis of patients who achieved and failed one- and three-year PFS/OS

Among patients who failed one-year PFS, those with grade 3 tumors were more common (20% versus 7%, *p* = 0.003), and those with an ECOG score of 0 were less common (40% versus 54%, *p* = 0.002). Achieving one-year PFS was more frequent in patients who received prior chemotherapy (51% versus 25%, *p* = 0.042) or hormone therapy (54% versus 20%, *p* = 0.008) than in those who did not. The frequency of receiving letrozole as additional hormone therapy was higher in patients who achieved one-year PFS compared to those who did not (77% versus 40%, *p* = 0.006). Median MTV (90.2 cm³ vs. 382.0 cm³, *p* = 0.016) and TLG (348.1 g vs. 1270.9 g, *p* = 0.026) were lower in patients who achieved one-year OS compared to those who did not. The proportion of patients with an ECOG score of 0 was higher among those who achieved one-year OS (55% vs. 23%, *p* < 0.001) (Table 2). Additionally, patients who achieved one-year PFS more frequently had a history of chemotherapy (51% vs. 25%) and previous endocrine therapy (54% vs. 20%) than those who did not (p-values 0.042 and 0.008, respectively). This was likely because most of the patients who received chemotherapy and prior endocrine therapy had an ECOG score of 0 (65% and 63% vs. 42% and 43%) and had metastatic disease after relapse rather than de novo metastasis (69% and 68% vs. 3% and 5%).

**Table 2:** Patient characteristics according to one-year PFS and OS

Variables	Patients who achieved one-year PFS (*n* = 67)	Patients who did not achieve one-year PFS (*n* = 20)	*p*-value	Patients who achieved one-year OS (*n* = 74)	Patients who did not achieve one-year OS (*n* = 13)	*p*-value
Age, mean ± SD	53 ± 9	59 ± 15	0.082	58 ± 15	60 ± 14	0.530
Ki-67, median (minimum-maximum)	15 (5–60)	20 (10–60)	0.117	15 (5–60)	20 (10–40)	0.194
Histopathological subtype, n (%)						
• IDC	53 (79)	15 (75)	0.455	57 (77)	11 (85)	0.422
• Others	14 (21)	5 (25)		17 (23)	2 (15)	
Grade, n (%),						
• Grade 1	6 (9)	0 (0)	**0.003**	6 (8)	0 (0)	0.058
• Grade 2	55 (82)	12 (60)		58 (78)	9 (69)	
• Grade 3	5 (7)	4 (20)		8 (11)	1 (8)	
• N/A	1 (2)	4 (20)		2 (3)	3 (23)	
TNM stage at diagnosis, n (%) n (%)						
• Stage 1	1 (2)	1 (5)	0.694	2 (3)	0 (0)	0.338
• Stage 2	11 (16)	3 (15)		13 (18)	1 (8)	
• Stage 3	12 (18)	5 (25)		16 (21)	1 (8)	
• Stage 4	43 (64)	11 (55)		43 (58)	13 (84)	
CDK 4/6 inhibitor agent, n (%)						
• Ribociclib	48 (72)	14 (70)	0.887	52 (70)	10 (77)	0.452
• Palbociclib	19 (28)	6 (30)		22 (30)	3 (23)	
When did the patient become metastatic? n (%)						
• De novo	42 (63)	11 (55)	0.357	42 (57)	11 (85)	0.070
• After relapse	25 (37)	9 (45)		32 (43)	2 (15)	
ECOG performance status, n (%)						
• 0	36 (54)	8 (40)	**0.002**	41 (55)	3 (23)	**< 0.001**
• 1	23 (34)	7 (35)		27 (37)	3 (23)	
• 2	7 (10)	0 (0)		5 (7)	2 (15)	
• 3–4	0 (0)	0 (0)		0 (0)	0 (0)	
• N/A	1 (2)	5 (25)		1(1)	5 (39)	
Metastatic areas, n (%)						
• Bone ± soft tissue	45 (67)	10 (50)	0.358	49 (66)	6 (46)	0.162
• Only visceral organs	3 (5)	1 (5)		4 (5)	0 (0)	
• Bone + visceral organs	19 (28)	9 (45)		21 (29)	7 (54)	
MTV (mL), median (minimum-maximum)	111.8 (0-3439.5)	90.4 (19.3-1757.7)	0.446	90.2 (0-3439.5)	382.0 (3.1-1613.6)	**0.016**
TLG (g), median (minimum-maximum)	520.1 (0-40125.1)	388.7 (48.8–9467.0)	0.593	348.1 (0-40125	1270.9 (11.2-6913.8)	**0.026**
Additional hormone therapy, n (%)						
• Fulvestrant	13 (19)	12 (60)	**0.006**	20 (27)	5 (39)	0.807
• Letrozole	52 (77)	8 (40)		52 (71)	8 (61)	
• Anastrozole	1 (2)	0 (0)		1 (1)	0 (0)	
• N/A	1 (2)	0 (0)		1 (1)		
Bone-modifying agent, n (%)						
• Denosumab	33 (49)	4 (20)	0.917	35 (47)	7 (54)	0.909
• Zoledronic acid	23 (34)	7 (35)		26 (35)	4 (31)	
• No	11 (17)	9 (45)		13 (18)	2 (15)	
Previous hormonal therapy, n (%)						
• Yes	36 (54)	4 (20)	**0.008**	39 (53)	8 (62)	0.389
• No	31 (46)	16 (80)		35 (47)	5 (38)	
Surgery history, n (%)						
• Yes	35 (52)	10 (50)	0.905	39 (53)	9 (69)	0.133
• No	32 (48)	10 (50)		35 (47)	4 (31)	
Adjuvant/neoadjuvant chemotherapy, n (%)						
• Yes	34 (51)	5 (25)	**0.042**	40 (54)	8 (62)	0.425
• No	33 (49)	15 (75)		34 (46)	5 (38)	
Radiotherapy, n (%)						
• Yes	45 (67)	10 (50)	0.263	27 (37)	9 (69)	0.540
• No	22 (33)	10 (50)		47 (53)	4 (31)	

*PFS* progression-free survival, *OS* overall survival, *SD* standard deviation, *IDC* invasive ductal carcinoma, *N/A* not available, *PR* progesterone receptor, *HER2* human epidermal growth factor receptor 2, *TNM* tumor, node, and metastasis

Patients who achieved three-year OS were younger on average (54 years versus 60 years, *p* = 0.043) and a higher proportion of having an ECOG score of 0 (78% versus 38%, *p* = 0.004) (Table 3).

**Table 3:** Patient characteristics according to three-year PFS and OS

Variables	Patients who achieved three-year PFS (*n* = 22)	Patients who did not achieve three-year PFS (*n* = 65)	*p*-value	Patients who achieved three-year OS (*n* = 27)	Patients who did not achieve three-year OS (*n* = 60)	*p*-value
Age, mean ± SD	59 ± 14	58 ± 15	0.848	54 ± 12	60 ± 15	**0.043**
Ki-67, median (minimum-maximum)	15 (4–35)	20 (5–60)	0.268	15 (4–60)	15 (5–60)	0.692
Histopathological subtype, n (%)						
• IDC	17 (77)	51 (78)	0.561	22 (82)	46 (77)	0.420
• Others	5 (23)	14 (22)		5 (18)	14 (23)	
Grade, n (%),						
• Grade 1	2 (9)	4 (6)	0.566	3 (11)	3 (5)	0.297
• Grade 2	18 (82)	49 (75)		22 (82)	45 (75)	
• Grade 3	2 (9)	7 (11)		2 (7)	7 (12)	
• N/A	0 (0)	5 (8)		0 (0)	5 (8)	
TNM stage at diagnosis, n (%) n (%)						
• Stage 1	0 (0)	2 (3)	0.803	0 (0)	2 (3)	0.779
• Stage 2	3 (14)	11 (17)		4 (15)	10 (17)	
• Stage 3	4 (18)	13 (20)		5 (18)	12 (20)	
• Stage 4	15 (68)	39 (60)		18 (67)	36 (60)	
CDK 4/6 inhibitor agent, n (%)						
• Ribociclib	13 (59)	49 (75)	0.119	17 (63)	45 (75)	0.186
• Palbociclib	9 (41)	16 (25)		10 (37)	15 (25)	
When did the patient become metastatic? n (%)						
• De novo	15 (68)	38 (59)	0.292	18 (67)	35 (58)	0.311
• After relapse	7 (32)	27 (41)		9 (33)	25 (42)	
ECOG performance status, n (%)						
• 0	16 (73)	28 (43)	0.066	21 (78)	23 (38)	**0.004**
• 1	4 (18)	26 (40)		6 (22)	24 (40)	
• 2	2 (9)	5 (8)		0 (0)	7 (12)	
• 3–4	0 (0)	0 (0)		0 (0)	0 (0)	
• N/A	0 (0)	6 (9)		0 (0)	6 (10)	
Metastatic areas, n (%)						
• Bone ± soft tissue	14 (64)	41 (63)	0.469	19 (70)	36 (60)	0.650
• Only visceral organs	0 (0)	4 (6)		1 (4)	3 (5)	
• Bone + visceral organs	8 (36)	20 (31)		7 (26)	21 (35)	
MTV (mL), median (minimum-maksimum)	101.9 (0-2047.4)	112.7 (3.1-3439.5)	0.922	72.8 (0-367.2)	127.6 (3.1-3439.5)	0.151
TLG (g), median (minimum-maksimum)	535.8 (0-13299.5)	381.8 (11.2-40125.1)	0.578	298.9 (0-1810.7)	561.7 (11.2-40125.1)	0.248
Additional hormone therapy, n (%)						
• Fulvestrant	3 (14)	22 (34)	0.230	5 (19)	20 (33)	0.360
• Letrozole	19 (86)	41 (62)		22 (81)	38 (63)	
• Anastrozole	0 (0)	1 (2)		0 (0)	1 (2)	
• N/A	0 (0)	1 (2)		0 (0)	1 (2)	
Bone-modifying agent, n (%)						
• Denosumab	7 (32)	31 (48)	0.954	12 (44)	30 (50)	0.706
• Zoledronic acid	11 (50)	23 (35)		11 (41)	19 (32)	
• No	4 (18)	11 (17)		4 (15)	11 (18)	
Previous hormonal therapy, n (%)						
• Yes	13 (59)	27 (42)	0.119	14 (52)	26 (43)	0.306
• No	9 (41)	38 (58)		13 (48)	34 (57)	
Surgery history, n (%)						
• Yes	11 (50)	33 (51)	0.549	13 (48)	31 (52)	0.442
• No	11 (50)	32 (49)		14 (52)	29 (48)	
Adjuvant/neoadjuvant chemotherapy, n (%)						
• Yes	13 (59)	26 (40)	0.096	15 (56)	24 (40)	0.132
• No	9 (41)	39 (60)		12 (44)	60 (60)	
Radiotherapy, n (%)						
• Yes	16 (73)	38 (58)	0.218	18 (67)	36 (60)	0.540
• No	6 (27)	27 (42)		9 (33)	24 (40)	

*PFS* progression-free survival, *OS* overall survival, *SD* standard deviation, *IDC* invasive ductal carcinoma, *N/A* not available, *PR* progesterone receptor, *HER2* human epidermal growth factor receptor 2, *TNM* tumor, node, and metastasis

**Fig. 3:**
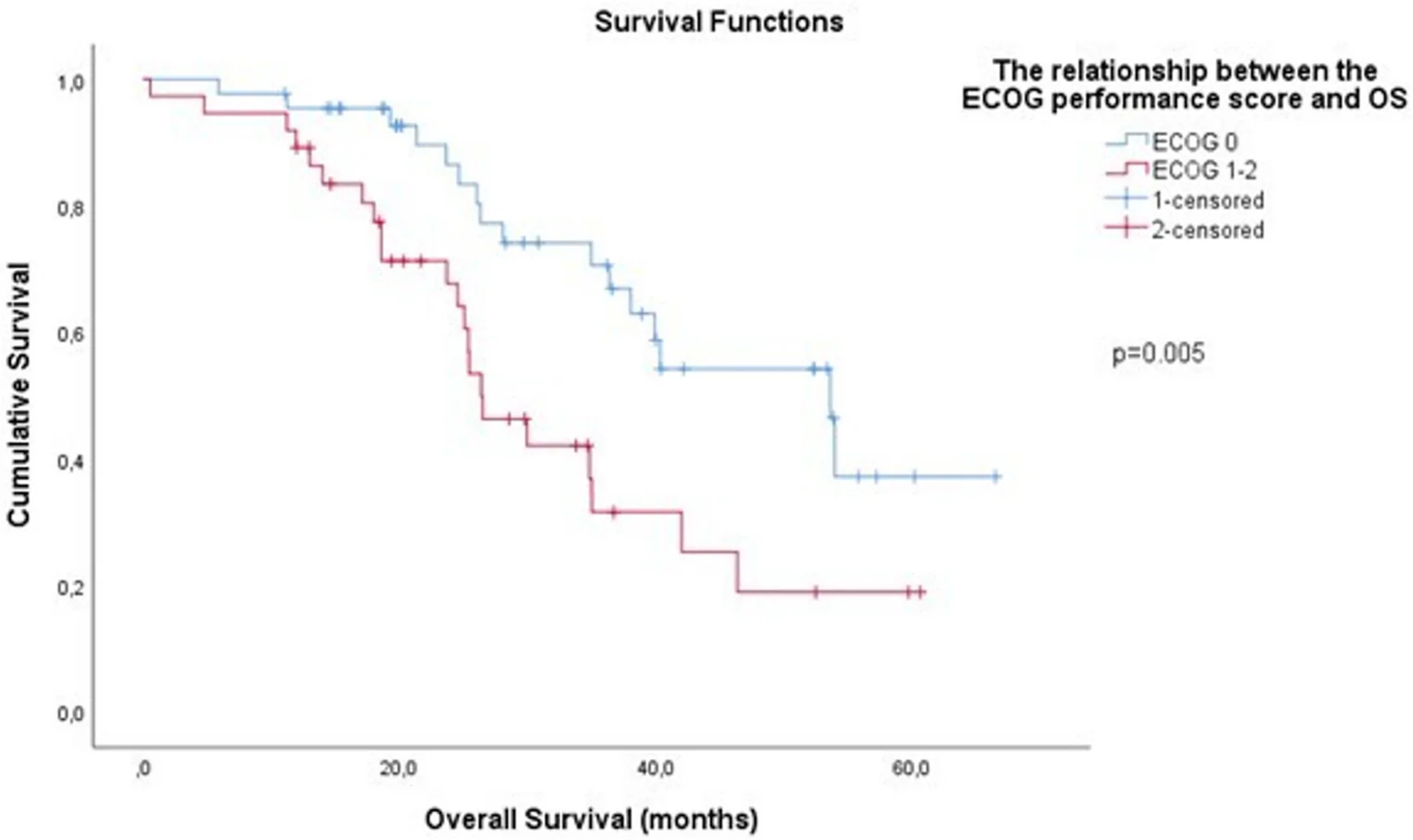
The relationship between the ECOG performance score and overall survival. The estimated median overall survival for patients with an ECOG score of 0 was 53.6 months (95% confidence interval, 39.8–67.4 months), while for those with an ECOG score of 1–2, it was 26.4 months (95% confidence interval, 20.9–31.8 months) (*p* = 0.005)

According to Kaplan-Meier analysis, there was no significant difference in the median estimated PFS between patients with an ECOG score of 0 and 1–2 (42.4 versus 31.0 months, *p* = 0.098). However, the estimated median OS for patients with an ECOG score of 0 was 53.6 months (95% confidence interval [CI], 39.8–67.4 months), while for those with an ECOG score of 1–2, it was 26.4 months (95% CI, 20.9–31.8 months) (*p* = 0.005) (Fig. 3).

### Prediction of PFS/OS using MTV and TLG

**Fig. 4:**
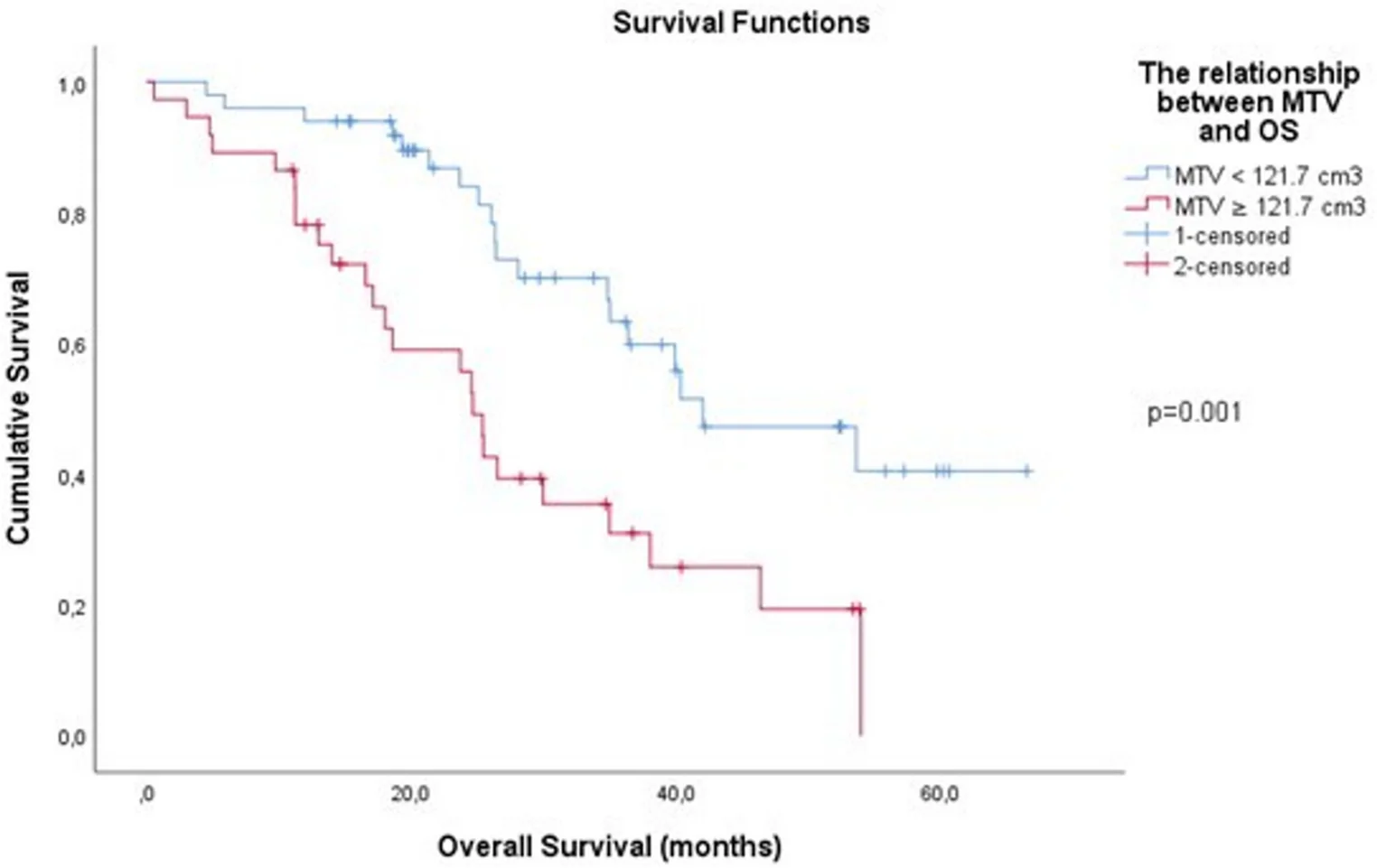
The relationship between MTV and overall survival. The median estimated overall survival was 42.0 months (95% confidence interval 25.8–58.2 months) for patients with MTV < 121.7 cm³ and 24.6 months (95% confidence interval 22.4–26.9 months) for patients with ≥ 121.7 cm^3^ (*p* = 0.001)

**Fig. 5:**
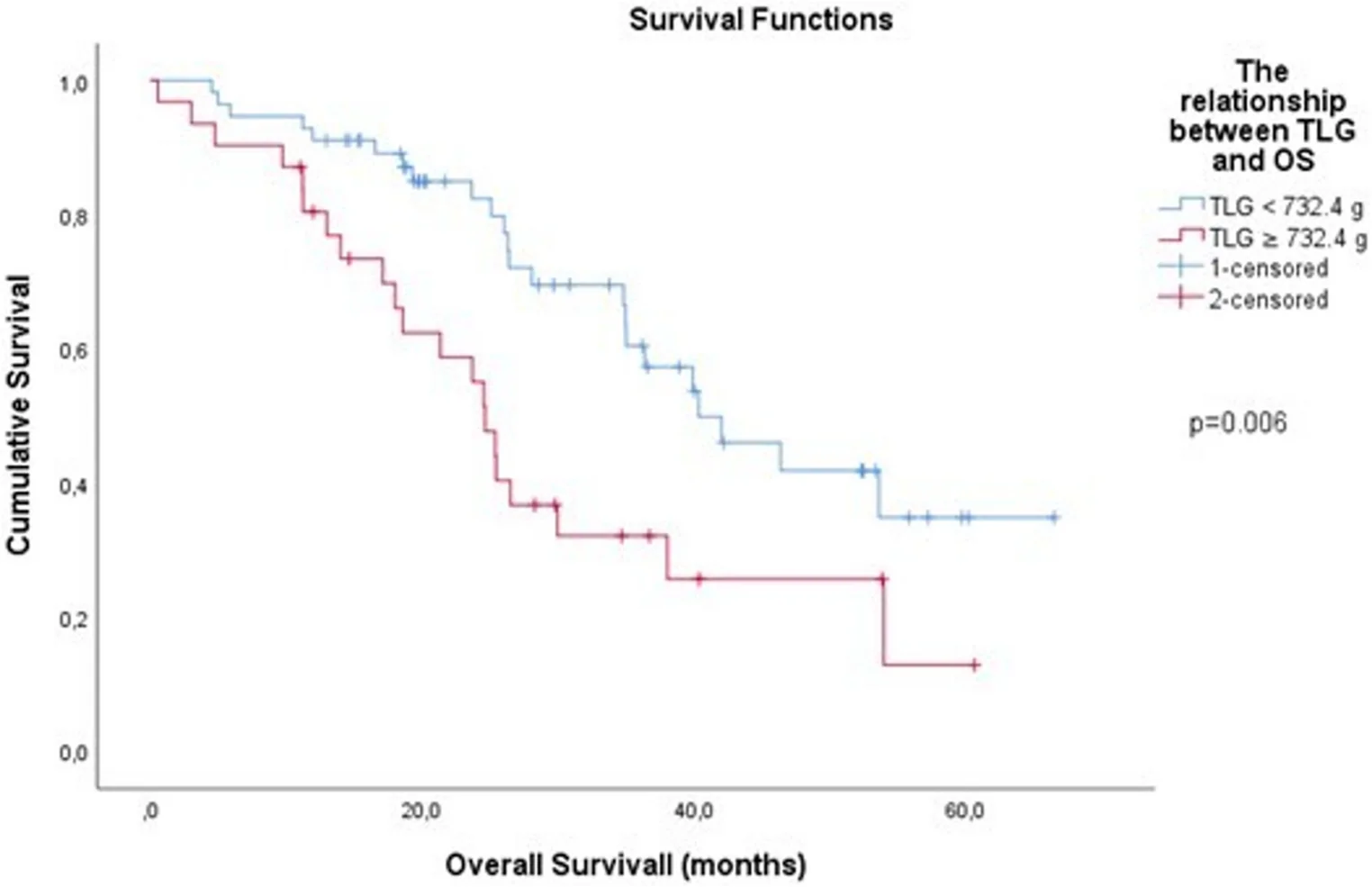
The relationship between TLG and overall survival. The median estimated overall survival was 40.3 months (95% confidence interval 29.5–51.2 months) for patients with TLG < 732.4 g and 24.6 months (95% confidence interval 21.8–27.4 months) for those with ≥ 732.4 g (*p* = 0.006)

The areas under the curve (AUC) for MTV and TLG in predicting progression were 0.570 and 0.559, respectively, with p values > 0.05. For predicting death, the MTV threshold was 121.7 cm³ with an AUC of 0.652 (*p* = 0.014), and the TLG threshold was 732.4 g with an AUC of 0.646 (*p* = 0.019). The median estimated OS was 42.0 months (95% CI 25.8–58.2 months) for patients with MTV < 121.7 cm³ and 24.6 months (95% CI 22.4–26.9 months) for patients with ≥ 121.7 cm^3^ (*p* = 0.001) (Fig. 4). The median estimated OS was 40.3 months (95% CI 29.5–51.2 months) for patients with TLG < 732.4 g and 24.6 months (95% CI 21.8–27.4 months) for those with ≥ 732.4 g (*p* = 0.006) (Fig. 5).

### Cox regression analysis results

Univariate and multivariate analyses showed that age ≥ 60 years (HR = 5.11, 95% CI 1.81–14.41, *p* = 0.002) and ECOG score > 0 (HR = 3.38, 95% CI 1.32–8.69, *p* = 0.011) are independent predictors of PFS (Table 4). Combination with letrozole appeared to be one of the predictors of PFS in the univariate analysis (HR = 0.325, 95% CI 0.18–0.59, *p* < 0.001), but since letrozole was the additional hormonal therapy agent in 69% of the patients included in the study, it was not included in the multivariate analysis to avoid bias. Neither variable served as an independent predictor of OS (Table 5). Neither MTV nor TLG was an independent predictor of PFS/OS in multivariate analyses (Tables 4 and 5).

**Table 4:** Univariate and multivariate cox proportional regression analysis of PFS

	Univariate analysis for PFS		Multivariate analysis for PFS	
Paremeter	HR (95% CI)	*P* value	HR (95% CI)	*P* value
Age ≥ 60	1.95 (1.06–3.57)	**0.027**	5.11 (1.81–14.41)	**0.002**
ECOG score > 0	1.68 (0.90–3.11)	0.102	3.38 (1.32–8.69)	**0.011**
Visceral metastasis	1.53 (0.86–2.72)	0.159	1.52 (0.72–3.22)	0.272
Previous surgery	1.24 (0.70–2.20)	0.469		
Adjuvant/neoadjuvant chemotherapy	0.45 (0.24–0.82)	**0.008**	0.49 (0.12–1.49)	0.209
Radiotherapy	0.50 (0.28–0.90)	**0.023**	0.32 (0.08–1.35)	0.121
Previous hormonal therapy	0.48 (0.27–0.88)	**0.015**	0.26 (0.05–1.30)	0.100
Bone-modifying agent	0.87 (0.41–1.87)	0.720		
Treatment with ribociclib	1.21 (0.64–2.31)	0.544		
Combination with letrozole	0.325 (0.18–0.59)	**< 0.001**		
De novo metastatic disease	0.65 (0.37–1.18)	0.160	0.83 (0.26–2.61)	0.749
Ki-67 ≥ 18	1.39 (0.76–2.58)	0.282		
Grade 2–3	1.33 (0.412–4.32)	0.618		
Histopathological subtypes other than IDC	1.10 (0.55–2.22)	0.786		
MTV ≥ 121.7 cm3	1.60 (0.89–2.83)	0.111	2.28 (0.97–5.37)	0.060
TLG ≥ 732.4 g	1.10 (0.61–1.99)	0.749		

*PFS* Progression-free Survival, *HR* Hazard ratio, *CI* Confidence Interval, *ECOG* Eastern Cooperative Oncology Group, *CDK* Cyclin Dependant Kinease, *IDC* Invasive Ductal Carcinoma, *MTV* Metabolic Tumor Volume, *TLG* Total Lesion Glycolysis

**Table 5:** Univariate and multivariate cox proportional regression analysis of OS

	Univariate analysis for OS		Multivariate analysis for OS	
Paremeter	HR (95% CI)	*P* value	HR (95% CI)	*P* value
Age ≥ 60	1.11 (0.61–2.01)	0.740		
ECOG score > 0	2.46 (1.28–4.72)	**0.006**	1.73 (0.85–3.50)	0.131
Visceral metastasis	1.84 (1.00-3.36)	0.052	1.80 (0.88–3.67)	0.108
Previous surgery	0.54 (0.29–0.99)	**0.043**	0.44 (0.13–1.50)	0.190
Adjuvant/neoadjuvant chemotherapy	0.87 (0.48–1.59)	0.659		
Radiotherapy	0.883 (0.46–1.68)	0.702		
Previous hormonal therapy	0.98 (0.54–1.77)	0.934		
Bone-modifying agent	0.85 (0.36–2.01)	0.705		
Treatment with ribociclib	1.15 (0.60–2.19)	0.678		
Combination with letrozole	0.61 (0.32–1.14)	0.133	0.53 (0.23–1.24)	0.144
De novo metastatic disease	0.60 (0.31–1.15)	0.114	1.88 (0.45–7.85)	0.384
Ki-67 ≥ 18	1.41 (0.75–2.64)	0.281		
Grade 2–3	1.25 (0.38–4.06)	0.705		
Histopathological subtypes other than IDC	1.05 (0.50–2.21)	0.895		
MTV ≥ 121.7 cm3	2.72 (1.49–4.96)	**0.001**	1.71 (0.63–4.66)	0.293
TLG ≥ 732.4 g	2.27 (1.25–4.11)	**0.008**	1.77 (0.63–4.94)	0.275

*OS* Overall Survival, *HR* Hazard ratio, *CI* Confidence Interval, *ECOG* Eastern Cooperative Oncology Group, *CDK* Cyclin Dependant Kinease, *IDC* Invasive Ductal Carcinoma, *MTV* Metabolic Tumor Volume, *TLG* Total Lesion Glycolysis

## Discussion

In this study, we examined the relationship between baseline 18 F-FDG PET/CT metabolic parameters and PFS/OS in patients with metastatic breast cancer treated with CDK 4/6 inhibitors plus endocrine therapy. We found that patients with MTV ≥ 121.7 cm³ or TLG ≥ 732.4 g had significantly shorter OS. However, in Cox regression analysis, MTV or TLG were not independent predictors of OS. OS was longer in patients with an ECOG score of 0; an ECOG score of 0 was an independent predictor of PFS, but not of OS. Moreover, age ≥ 60 years was also an independent predictor of PFS.

Lasnon C et al. investigated the predictive value of 18-month PFS in 66 patients with metastatic breast cancer treated with ER+/HER2-, CDK 4/6 inhibitors plus endocrine therapy, using metabolic parameters measured at baseline and after 18 F-FDG PET/CT posttreatment, along with changes in these parameters [17]. Only patients with a baseline Dmax (distance between the two most distant lesion centers) of 18.10 cm or less had significantly better 18-month PFS survival than the others. Baseline SUVpeak, MTV, TLG, or post-treatment changes in these parameters did not predict 18-month PFS; similarly, we found that MTV or TLG did not predict PFS in our study. The authors emphasized that although some studies find SUV, MTV, and TLG to be predictive in breast cancer, their study’s contradictory results may be due to ER+/HER2- breast cancer. OS was not a focus in this study, and the impacts of MTV or TLG on OS were not examined.

Kubeczko M et al. reported that in 39 patients with metastatic breast cancer treated with CDK 4/6 inhibitors plus endocrine therapy, median PFS was longer in patients with SUVmax of the most hypermetabolic lesion < 8.4 compared to those with ≥ 8.4 (30.7 months versus 13.0 months, *p* = 0.038), but this was not true for OS [15]. Cox regression analysis identified poor prognostic factors for PFS as an ECOG score of 2 (compared to 0 and 1) -in our study, an ECOG score greater than 0 was a predictor for PFS-, recurrent disease (compared to de novo metastasis) -which was not a predictor of PFS in our study-, and SUVmax ≥ 8.4 (compared to < 8.4). SUVmax is an easy parameter to calculate, but its reliability is limited because it is influenced by many factors related to the patient, the radionuclide injection process, imaging, and image processing. A significant limitation of this study is that the prognostic roles of MTV and TLG, which indicate tumor burden, were not investigated. Additionally, the small number of patients (*n* = 39) and the short median follow-up period (16.9 months) may have contributed to the inability to identify any prognostic factors related to OS.

Annovazzi A et al. treated 114 patients with HR+/HER2- advanced or metastatic breast cancer using CDK 4/6 inhibitors in combination with endocrine therapy, aiming to determine prognosis based on 18 F-FDG PET/CT in these patients [16]. They showed that PFS was longer in patients with a post-treatment Deauville score ≤ 3 (42.0 versus 21.0 months, *p* = 0.008) and a baseline MTV ≤ 31.4 cm³ (42.0 versus 18.0 months, *p* = 0.0026). They proposed that combining the post-treatment Deauville score with baseline MTV could help in prognostic stratification. They also found that ECOG score, second- or third-line CDK 4/6 inhibitor use, and the presence of liver metastases were associated with OS. In our study, higher MTV was not associated with poor PFS/OS, and ECOG score was associated with poor OS but was not an independent predictor.

Li Y et al. investigated the predictive power of metabolic parameters from baseline 18 F-FDG PET/CT before initial treatment to predict PFS in 177 patients with HR+/HER2-, HER2+, and triple-negative metastatic breast cancer [19]. In an analysis including all patients, higher baseline SUVmax, SUVmean, MTV, TLG, and heterogeneity index (which is the highest SUVmax divided by the lowest SUV among all metastases) were associated with shorter PFS. In HR+/HER2- and HER2 + patients, only the heterogeneity index was associated with poorer PFS. In triple-negative patients, SUVmax, MTV, and TLG were associated with poorer PFS. The authors highlighted that parameters indicating tumor burden in triple-negative breast cancer are likely to have prognostic importance due to rapid proliferation and spread. In our study, high MTV and high TLG were associated with poorer OS, but these factors were not independent predictors in multivariate Cox regression analysis. It’s not surprising that high tumor burden correlates with shorter OS, but tumor burden might not be the most critical factor influencing OS. An unexpected finding was the lack of a significant association between MTV/TLG and PFS. This finding may be attributable, at least in part, to the methodology used to determine disease progression. In this study, disease progression was identified by reviewing patients’ medical records. Detection of disease progression may have been delayed because follow-up imaging modalities were not standardized across patients (for example, sclerotic bone metastases that look stable on CT but have significant metabolic activity cannot be detected without PET/CT imaging). Consequently, some patients with earlier disease progression may have been misclassified as having longer PFS, thereby masking the effect of MTV/TLG on PFS. In addition, PET/CT imaging was limited to the vertex-to-mid-thigh field of view. Therefore, potential metastatic sites outside the imaging range (for example, distal femur and tibial metastases) were not included in the MTV and TLG calculations. Consequently, omission of tumor burden outside the imaging field may have resulted in underestimation of the true MTV and TLG values, particularly in patients with extensive skeletal metastases, and as a result, the effect of MTV/TLG on PFS or OS may be masked. The possibility that sclerotic metastases, which are known to exhibit low 18 F-FDG avidity, could have been present in the patient group in this study may have complicated the assessment of true tumor burden. These methodological limitations may have reduced the ability of MTV and TLG to emerge as independent prognostic factors. ECOG score and age have prognostic significance in metastatic breast cancer. In HR+/HER2- patients with advanced breast cancer treated with palbociclib plus fulvestrant or letrozole, an ECOG score of > 0 was found to be an independent predictor of OS, but age of ≥ 65 years did not predict OS [20]. In patients with triple-negative breast cancer and brain metastases, an ECOG score of ≥ 2 was found to be associated with worse survival [21]. In women with breast cancer and leptomeningeal metastasis, age ≥ 60 years and ECOG score 2–4 were associated with poorer survival [22]. However, some studies have indicated that age is not an independent predictor of survival in HR+/HER2- metastatic breast cancer [15–17]. In our study, age > 60 years (HR = 5.11, 95% CI 1.81–14.41) and ECOG score > 0 (HR = 3.38, 95% CI 1.32–8.69) were independent risk factors for PFS, but not for OS. We believe that the ECOG score, rather than age, may serve as a better predictor of OS.

It is known that the prognosis is poorer in grade 3 breast cancer [23, 24]. However, we found no studies in the literature examining the prognostic significance of primary tumor grade in metastatic patients. In our study, patients with grade 3 primary tumors were more commonly observed among those who did not achieve one-year PFS (20% versus 7%, *p* = 0.003).

Our study has some limitations. The single-center retrospective design represents the principal limitation of this study. Although 51% of patients died and 54% experienced disease progression during follow-up, the number of events remained relatively limited for robust survival analyses. However, the median follow-up period of 25.4 months is reasonable for a study involving metastatic patients. Our goal was to evaluate the prognostic value of MTV and TLG in metastatic breast cancer patients receiving CDK 4/6 inhibitors. As discussed above, 18 F-FDG PET/CT imaging was performed from the vertex to the mid-thigh, potentially excluding metastatic lesions located outside the imaging field. Consequently, the calculated MTV and TLG values may not have fully reflected the true whole-body tumor burden. This limitation may have contributed to the lack of independent prognostic significance of MTV and TLG for PFS and OS in the multivariate analyses.

In conclusion, baseline 18 F-FDG PET/CT may provide prognostic insights in HR+/HER2- metastatic breast cancer treated with CDK 4/6 inhibitors and endocrine therapy. High MTV and TLG were associated with poorer OS in univariate analyses, suggesting that patients with elevated MTV and TLG may benefit from closer clinical surveillance. Among the clinical variables evaluated, an ECOG score > 0 was associated with poorer OS and emerged as an independent predictor of PFS. Age ≥ 60 years is also an independent predictor of PFS.

## Data Availability

The datasets generated during and/or analysed during the current study are available from the corresponding author on reasonable request.
